# Evaluation of sense-strand mRNA amplification by comparative quantitative PCR

**DOI:** 10.1186/1471-2164-5-76

**Published:** 2004-10-06

**Authors:** Loyal A Goff, Jessica Bowers, Jaime Schwalm, Kevin Howerton, Robert C Getts, Ronald P Hart

**Affiliations:** 1 W.M. Keck Center for Collaborative Neuroscience, Rutgers University, Piscataway, NJ 08854 USA; 2 Genisphere, Inc., Hatfield, PA 19440 USA

## Abstract

**Background:**

RNA amplification is required for incorporating laser-capture microdissection techniques into microarray assays. However, standard oligonucleotide microarrays contain sense-strand probes, so traditional T7 amplification schemes producing anti-sense RNA are not appropriate for hybridization when combined with conventional reverse transcription labeling methods. We wished to assess the accuracy of a new sense-strand RNA amplification method by comparing ratios between two samples using quantitative real-time PCR (qPCR), mimicking a two-color microarray assay.

**Results:**

We performed our validation using qPCR. Three samples of rat brain RNA and three samples of rat liver RNA were amplified using several kits (Ambion messageAmp, NuGen Ovation, and several versions of Genisphere SenseAmp). Results were assessed by comparing the liver/brain ratio for 192 mRNAs before and after amplification. In general, all kits produced strong correlations with unamplified RNAs. The SenseAmp kit produced the highest correlation, and was also able to amplify a partially degraded sample accurately.

**Conclusion:**

We have validated an optimized sense-strand RNA amplification method for use in comparative studies such as two-color microarrays.

## Background

One of the principal complications in microarray analysis of gene expression is the relatively large amount of input RNA required for each assay. On average, 1–20 μg of total RNA are required per study using glass microarrays [1-4]. This is easily obtained from standard tissue samples, but is more difficult to obtain from smaller samples, such as laser capture microdissections [5,6]. The primary impediment to the use of laser capture microscopy (LCM) in gene expression analysis is that microdissections yield insufficient mRNA due to low total RNA recovered from small sample sizes. With samples such as these, the ability to conduct a linear amplification of the target mRNA becomes imperative, to ensure that enough material is available for gene expression analysis. There are several methods for amplifying RNA including the arithmetic transcription methods [7,8], PCR based exponential amplification, or a combination of both arithmetic and exponential amplification [9]. Each method has proven effective in generating large amounts of amplified RNA from small starting samples. Each method is not without its drawbacks however. PCR based amplification has been shown to amplify sequence-dependent biases geometrically, and hybridization kinetics during the thermal cycles can create sequence-dependent and abundance-dependant biases [10]. New methods must be carefully validated with large numbers of mRNAs before they may be accepted for general use.

Most RNA amplification methods are based on the T7-based antisense RNA amplification technique first described by Van Gelder and Eberwine in 1990 [7]. In this technique poly(A)^+ ^mRNA is reverse-transcribed and converted into double stranded cDNA using an oligo(dT) primer containing a promoter for T7 RNA polymerase. The second strand cDNA serves as a transcription template for amplified antisense RNA (aRNA) production. cDNA microarray studies using T7 amplified RNA have shown that the technique yields reproducible results that correlate with the results obtained from using total RNA [2,9,11]. This method is incompatible, however, with standard spotted oligonucleotide microarrays when combined with conventional reverse transcription based labeling methods.

Spotted oligo microarrays consist of 'long' 50–80 mer *sense-strand *oligonucleotide probes arrayed onto a suitable substrate. Each probe sequence is designed to hybridize to a specific antisense cDNA reverse transcribed from a given mRNA species. The advantage of spotted oligo microarrays over cDNA microarrays is that the oligos can be designed to be more specific, with similar hybridization kinetics, lower homology among related transcript probes, and selection among different splice variants of the same gene. However, aRNA prepared from Eberwine-amplified mRNA would produce a sense-strand cDNA target that would not hybridize with sense-strand oligo probes on the microarray.

The Genisphere SenseAmp linear mRNA amplification method produces sense-strand amplified mRNA by incorporating a double stranded T7 promoter into the 3' end of the first strand cDNA, driving transcription of an amplified RNA with the same strandedness as mRNA. SenseAmp linear amplification also allows for the use of dT and random primers in the synthesis of cDNA. This variation on the amplification protocol may be as effective on partially degraded mRNA or, using random primers in a first-strand reaction, on RNAs lacking a poly(A) tail. Further, the use of random primers combined with dT priming may help to reduce the 3' bias associated with Eberwine-based amplification methods [12,13] by improving the access of reverse transcription to the 5' end of mRNA.

Most studies evaluating amplification validity compare unamplified to amplified material [3,14-17]. However, this is not a good model of experiments normally performed with spotted oligo microarrays. In most two-color microarray experiments, an experimental sample is compared with a reference sample on the same microarray, so it is the ratio between two samples that becomes the most important parameter. RNA amplification may have some sequence bias, but as long as the bias is consistent between reactions, the effect of the bias may be canceled. We chose, therefore, to evaluate amplification strategies by comparing two RNA samples both before and after amplification, and correlating the ratios obtained before and after amplification. This key difference allows us to identify the best amplification method for use with two-color microarrays.

Throughout the course of this study, several pre-production versions of SenseAmp were evaluated with the aim of judging the optimal method. Total RNA from replicate rat brain and liver samples was amplified using one of several different techniques including three versions of the SenseAmp method, MessageAmp from Ambion, Nugen's Ovation RNA-based single primer isothermal amplification (Ribo-SPIA) method, and as an additional study, SenseAmp amplification of partially degraded RNA samples. The ratio of amplified RNAs obtained from each method was compared via relative qPCR to that of unamplified mRNAs from the same pool to determine how accurately the relative abundances were preserved. The use of qPCR provides a much broader range of results than possible with microarrays [18]. Relative qPCR analysis also allowed for the quantification of amplified RNA regardless of which strand was amplified, thus a direct comparison could be made between the various amplification techniques.

The fidelity of the amplification methods was determined using the ΔΔC_t _relative quantification method for qPCR. This method is used to compare the expression of a given gene in one sample relative to a second, and is based on the amplification efficiency of the PCR primer pairs used [19]. Quantitative PCR was selected because of its universal use as a microarray validation method [10,11,18-21], enhanced dynamic range [18-20], and ease of use with limited sample sizes for evaluating expression changes for several hundred genes. The basis of this method is the assumption that the exponential amplification of the starting product, and therefore the amount of PCR products produced with each round of amplification, is dependent upon the efficiency of each PCR primer pair. This efficiency is determined either experimentally or is calculated from the raw fluorescence data obtained during the qPCR amplification [22]. Equation (1) was used to compare the expression of 192 different genes in rat liver and rat brain samples. Triplicate total RNA samples from rat brain and liver were analyzed for each pair of primers targeting the mRNA concentrations of a given gene.

*Ratio of gene expression = E*^-ΔΔ*Ct *^    (1)

Through comparison of the relative gene expression across the various different amplification techniques, we were able to determine that each amplification method produces amplified RNA in quantities that accurately reflect the original mRNA proportions. The SenseAmp kit exhibited the best correlation to the unamplified control, and was effective in amplifying degraded RNA samples as well. In addition, we inadvertently identified a potential bias that can arise with the use of the oligo dT in reverse transcription priming.

## Results

**Figure 1:**
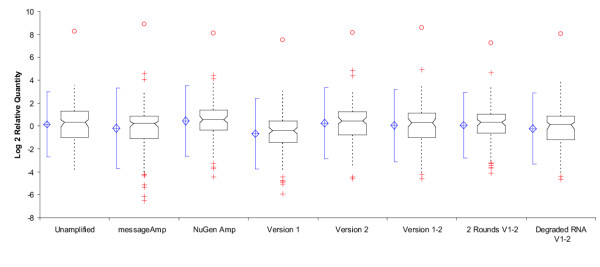
Distributions of liver/brain RQ ratios for all amplification methods. Box and whiskers plot showing the distribution of log_2 _RQ ratios for each amplification method. The blue diamond is centered on the mean and shows the 95% CI of the mean. The blue lines depict the percentile range. The center of the notched box is the median, with the notches showing the 95% CI of the median. The boxes show the inter-quartile range (IQR). Dashed lines are 1.5 times the IQR. Outliers are shown as red crosses (1.5–3.0 times the IQR) or red circles (>3.0 times the IQR).

**Figure 2:**
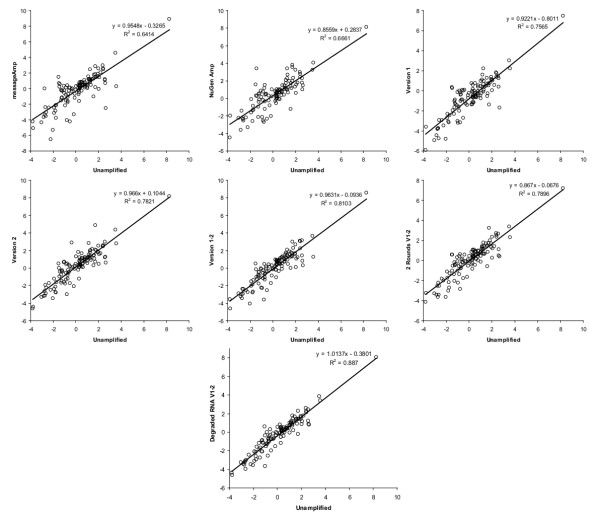
Scatterplots comparing liver/brain log_2 _RQ ratios of amplified RNAs with unamplified RNA. For each amplification method, a scatter plot shows the correlation of the liver/brain ratio to that of unamplified RNA. A linear regression fit is plotted as a line with the equation shown. The coefficient of determination (R^2^) corresponds to the square of the correlation coefficient (r) in Table 1.

We compared liver/brain expression ratios for a broad collection (n = 192) of mRNAs before and after amplification. Rat brain and liver RNAs were chosen since we needed to begin with large quantities of unamplified materials in order to test several amplification reactions on the same starting material, and to reliably assay the unamplified RNA. Several variations on the amplification method were tested to determine which method best replicated the distribution of liver/brain ratios found in unamplified RNAs. As anticipated, each of the amplification techniques produced amplified RNA that reproduced the full range of relative quantities (RQ) between liver and brain RNAs (Figure 1) and correlated well with the initial mRNA pool (Table 1, Figure 2). The SenseAmp version 1–2, which was designed to incorporate aspects of both version 1 and version 2, was shown to be most similar to the unamplified control results with a correlation coefficient of 0.90. As indicated by the lack of overlap in the 95% confidence intervals, SenseAmp version 1–2 produced amplified RNA with greater fidelity than either the MessageAmp or Ovation methods. Furthermore, each successive version of the SenseAmp protocol appeared to enhance the fidelity of the result. A series of two rounds of amplification with SenseAmp version 1–2 was indistinguishable in correlation to the unamplified control from a single round. These results suggest that each amplification technique is capable of producing linearly-amplified RNA that represents the relationships of the two original tissues. The SenseAmp kit (version 1–2) produced the most accurate reproduction of the original liver/brain ratios while also providing a sense-strand amplified RNA appropriate for use with oligonucleotide microarrays.

**Table 1:** Correlations between liver/brain RQ ratios of amplified vs. unamplified RNAs. For each correlation, n is the number of PCR results retained after filtering the amplification efficiency [22]. The correlation coefficient (r) is shown along with its 95% confidence interval (CI). Each correlation was significant at p < 0.0001. A cross-correlation matrix showing all relationships between samples is available at

	n*	***r***^‡^	***95% CI***	***p***
***MessageAmp™***	121	0.80	0.74 To 0.85	<0.0001
***Ovation™***	112	0.82	0.76 To 0.86	<0.0001
***SenseAmp™ Version 1***	118	0.87	0.83 To 0.90	<0.0001
***SenseAmp™ Version 2***	121	0.88	0.85 To 0.91	<0.0001
***SenseAmp™ Version 1–2***	121	0.90	0.87 To 0.93	<0.0001
***2 Rounds Version 1–2***	121	0.89	0.85 To 0.92	<0.0001
***SenseAmp™ on degraded RNA***	121	0.94	0.92 To 0.96	<0.0001

* *number of valid samples shared with unamplified control*

^‡^*Pearson correlation coefficient*

Amplification of partially-degraded RNA samples using the SenseAmp version 1–2 method also produced a high correlation of liver/brain ratios to those from unamplified RNAs (r = 0.94). This high correlation using degraded RNA appeared to be due to the presence of random primer in the amplification reaction. Comparing the rank order of mRNA abundances in liver (Table 2), we found that reactions using oligo dT primers generally produced higher correlations between amplified RNAs indicating that a similar offset to the rank order was occurring for all dT based methods. However comparing the dT primer rank order to that of random primed samples demonstrates greater dissimilarity, although this effect was variable. We interpret these results as supporting the hypothesis that the addition of a random primer to the amplification assay inadvertently enhances the correlation to random-primed, unamplified RNA, partially offsetting the negative effect of RNA degradation.

**Table 2:** Rank correlations of liver C_t _values identifies effects of oligo d(T) primers vs. random hexamer primers.

	**Unamplified**	*MessageAmp*	*NuGen*	*Version 1*	*Version 2*	*Version 1–2*	*2 Rounds V 1–2*
*MessageAmp*	***0.75***	*1*					
*NuGen*	***0.65***	*0.83*	*1*				
*Version 1*	***0.61***	*0.68*	*0.61*	*1*			
*Version 2*	***0.84***	*0.92*	*0.78*	*0.71*	*1*		
*Version 1–2*	***0.84***	*0.90*	*0.84*	*0.67*	*0.95*	*1*	
*2 Rounds V1–2*	***0.79***	*0.89*	*0.81*	*0.67*	*0.93*	*0.92*	*1*
**Degraded V1–2**	**0.85**	***0.85***	***0.77***	***0.71***	***0.89***	***0.91***	***0.85***

Rank cross-correlation matrix for liver C_t _values. The mean cycle threshold (C_t_) values obtained with liver RNA samples were rank-ordered and correlated. Results indicate the faithful reproduction of an ordered list of mRNA concentrations in liver RNA before and after amplification. Results were filtered for acceptable PCR efficiencies (see Methods), producing 138 primer pairs for this analysis. Methods using random primer (including unamplified RNA) are bold, those using oligo d(T) are italicized. Scatter plots of each rank C_t _correlation are available at:

## Discussion

Most studies of the fidelity of amplified RNA have compared the amplified sample to the original total RNA sample exclusively [2-4,10,14]. While this is valid, the approach described here more accurately reproduces the standard experimental conditions for two-color microarray expression analysis by comparing the ratio of gene expression between two different samples. The ratios obtained for amplified brain RNA vs. amplified liver RNA are then compared to the ratios from the unamplified control comparison (Table 1, Figure 2). Any reproducible biases within the techniques are represented in both the brain and liver samples and therefore cancelled out in the comparison. This approach models a two-color gene expression comparison experiment and demonstrates the differences in expression profiles obtained from different amplification techniques.

Using this approach, all three amplification kits tested had correlation coefficients of 0.80 or greater, indicating a great deal of fidelity in amplifying paired samples of RNA. The SenseAmp kit performed relatively better among the three, with a correlation coefficient of 0.90, with the 95% confidence interval lying above the means and intervals produced with MessageAmp or Ovation methods. Other researchers have shown that additional rounds of amplification yield reproducible results for a single RNA sample with only modest biases [6,10]. The fidelity of amplification was maintained in the course of our experiments with the SenseAmp production kit, through a second round of amplification. The similar correlations between the SenseAmp version 1–2 and the two rounds of version 1–2 amplification indicate that a second round of amplification does not significantly affect the relative abundance of mRNA. Of the three SenseAmp versions tested, version 1–2 had the highest correlation to unamplified RNA using this approach. In order to confirm that qPCR was an appropriate choice for validating RNA amplification procedures, we compared the brain vs. liver expression ratios on oligonucleotide arrays of SenseAmp version 1–2 amplified to unamplified RNA (not shown). As we observed with qPCR, a high correlation of about 0.93 was observed after comparing about 2700 differentially expressed genes, indicating that our qPCR experimental design was appropriate.

Variations in correlation among the versions of the SenseAmp method may be due to modifications to the structure of the T7 promoter/cDNA template. The version 1 template consisted of a completely double stranded linear DNA molecule, with one strand of the promoter synthesized enzymatically. For version 2, the T7 promoter was composed of two prehybridized, synthetic DNA strands ligated to a single stranded cDNA template. For version 1–2, a double stranded T7 promoter was synthesized onto the end of a single stranded cDNA template from a T7 template oligo by a 3' recessed end "fill-in" reaction using the Klenow fragment of DNA polymerase I. Like version 1, the promoter contains one enzymatically-synthesized strand and like version 2 the cDNA portion is single stranded. The incorporation of an enzymatically-synthesized strand appears be a more effective initiation site for the T7 polymerase (unpublished results). Furthermore, single stranded DNA templates downstream of the ds T7 promoter have been shown to be very efficient T7 polymerase templates [23,24] demonstrating a 2 fold improvement in kinetics [24]. The combined increase in T7 amplification efficiency in version 1–2 may preserve the distribution of mRNA concentrations in the amplified product.

Previous studies have cautioned against comparing samples using different reverse transcription primers [2]. Priming with oligo dT reduced PCR yields and created 3' and sequence-specific biases compared with the use of random primer [13,14,25]. These biases arise from the specificity shown by oligo dT primer for the 3' poly(A) tail and low processivity of T7 polymerase, as well as the presence of internal poly(A) sequences which may act as additional priming sites for the oligo dT. Random primer has also been shown to create a better 3'/5' ratio than the oligo dT primer [14].

Our experimental design called for the use of oligo dT primer in most of the RNA amplification reactions but random primer in the cDNA synthesis phase of qPCR. This design was used for each of the comparison experiments and therefore any bias introduced by the oligo dT primed reaction would be repeated for each of the amplification techniques. The effect of the oligo dT priming in the RNA amplification was identified when the degraded RNA sample was amplified using a mixture of oligo dT primer and random primer. The result showed that the degraded sample amplification resulted in a higher correlation to the unamplified control than any of the amplification techniques including the SenseAmp amplification of intact RNA. It is our interpretation that this high correlation of the amplified, degraded samples to the unamplified qPCR samples may be due to the common use of random primers for both data sets.

## Conclusions

Overcoming the problem of tissue heterogeneity with LCM and other, similar techniques will allow the research community to focus its efforts on the biologically relevant cell types. The use of RNA amplification with these small, cell-type specific techniques provides reliable and reproducible quantities of mRNA suitable for high-throughput gene expression profiling. Amplification from small amounts of LCM-selected samples provides stronger hybridization signal and reduced biological noise attributed to the presence of other cell types.

RNA amplification has been shown here, and elsewhere, to be both a useful and consistent technique for production of practical amounts of RNA when limited starting material is available. While there are several reliable amplification methods available, most amplify an antisense RNA which is suitable for cDNA microarray analysis. The most apparent benefit of the SenseAmp method is the amplification of the sense mRNA strand. This allows for the direct use of cDNA reverse-transcribed from amplified RNA as a hybridization target for oligo microarrays, and any other analysis that requires a sense-strand orientation. In addition, we observed similar liver/brain ratios between amplified RNAs and unamplified RNAs. This comparison models the relative expression ratios observed with two-color microarrays. While each of the methods tested produced acceptable results, the SenseAmp methods provided optimal correlation between unamplified samples and sense-strand amplified RNA.

## Methods

### Primer design

A subset of 192 sequence targets was chosen from the Compugen/Sigma-Genosys Rat 8 K oligo library for qPCR analysis. Using previously-analyzed microarray results as a guide (not shown), we selected targets with a broad range of expression ratios from brain-specific, through common, to liver-specific mRNAs. GAPDH mRNA was also selected for normalization. Primers were designed for all 193 sequences using Applied Biosystems Primer Express software v2.0 (Applied Biosystems, Foster City, CA). Primers were designed to have a T_m _between 58°C and 60°C and with an optimal length of 20 nt. The %GC content was held between 20% and 80% with no 3' GC clamp. The target amplicon for each sequence was designed to be between 50 and 150 nt with an optimal T_m _of 85°C. The target mRNAs represented a broad range of sizes (as measured by cDNA lengths; range 110–8074; mean 1876 nt; 190 nt 95% CI) and base composition (range 38–68% GC; mean 52% GC; 0.85% 95% CI). Amplicons were distributed between 5'UTR (8.7%), coding sequence (82.0%), and 3'UTR (9.2%). Primers were purchased from Sigma-Genosys (The Woodlands, TX). The final working concentration for each of the primer pairs was 50 nM. A table of target sequences and primers is available in the supplemental materials .

### Preparation of total RNA

Samples of rat brain (n = 3) and rat liver (n = 3) were frozen in liquid nitrogen and ground to a coarse powder. RNA was isolated from each sample using TRIzol (Invitrogen, Carlsbad CA). After isolation, samples of the prepared RNA were further purified using a Qiagen RNeasy column (Qiagen, Valencia CA). Total RNA was quantified by UV spectrophotometry and the integrity was assessed using a Bioanalyzer model 2100 (Agilent, Palo Alto CA). Identical total RNA samples were divided among the 8 different experiments (including unamplified control).

Degraded RNA was prepared by treatment at 65°C for 15 minutes in fragmentation buffer (40 mM Tris acetate, pH 8.1, 100 mM potassium acetate, 30 mM magnesium acetate). Samples of RNA degraded under these conditions were analyzed on an Agilent Bioanalyzer using RNA Nano chips and 2100 Expert software. Degraded samples typically had little or no 28S rRNA peak remaining and a broad smear below a weaker 18S rRNA peak, corresponding to an RNA Integrity Number (RIN) of 4.9 (using baseline correction) out of a score of 10 for RNA of ideal quality.

### RNA amplification

Each of the three replicates for each tissue was amplified using the Ambion messageAmp (Ambion, Austin TX), NuGen Ovation AminoAllyl amplification (NuGen Technologies, San Carlos CA), or SenseAmp version 1, version2, or version 1–2 kits. SenseAmp, version 1–2, is the commercial version of the Genisphere SenseAmp kit. Amplification with each method was done according to the protocol outlined for each method. For each of the MessageAmp and SenseAmp amplifications, 0.75 μg of input total RNA was used. For SenseAmp with random priming, 250 ng of input total RNA was used. The final yield of amplified RNA for each method was ~36 μg. For the NuGen Ovation amplification, 70 ng of input total RNA was used to yield ~10 μg of amplified cDNA.

For all versions of SenseAmp, total RNA was reverse transcribed using 100 ng of an anchored dT primer [d(T)_24_V] as described in the SenseAmp manual . For the degraded RNA samples, random 9 mers were added to the reverse transcription reaction at twice the mass of the input total RNA (e.g. 500 ng random primers per 250 ng of total RNA). Superscript II (Invitrogen) was used for all reverse transcriptions. The cDNA was purified using a MinElute PCR Purification Kit (Qiagen).

#### SenseAmp version 1

The purified cDNA was 3' tailed with dATP. A T7-promoter/oligo d(T) primer was used to initiate second strand cDNA synthesis using *E. coli *DNA polymerase I (Invitrogen) at 16°C for 2 hours as described by the manufacturer. Double-stranded cDNA was purified using the MinElute PCR Purification Kit and used for *in vitro *transcription using the MegaScript kit (Ambion). Amplified sense RNA was purified using the RNeasy Kit (Qiagen) and the manufacturer's recommendation for RNA clean up.

#### SenseAmp version 2

The purified cDNA was poly d(T) tailed as described in the SenseAmp (Genisphere) product manual. Excess double-stranded T7-promoter was ligated to the 3' poly d(T) tail on the cDNA in 1X ligation buffer (Roche) at room temperature for 30 minutes. The double stranded (ds)T7 promoter consisted of equal molar amounts of a T7 promoter oligo hybridized to a complementary oligo have a 10 base d(A)_10 _overhang on the 3' end prehybridized in 6X ligation buffer (Roche Applied Science, Indianapolis IN). Excess unligated ds T7 promoter was removed using the MinElute PCR Purification Kit. The purified cDNA, which contained a double-stranded T7 promoter linked to a single stranded cDNA template [23,24], was used for *in vitro *transcription using the MegaScript kit (Ambion). Amplified sense RNA was purified using the RNeasy Kit (Qiagen).

#### SenseAmp version 1–2

The complete process is described in the Gensiphere SenseAmp product manual. Briefly, the purified cDNA was poly d(T) tailed. A T7-promoter/oligo dA template strand with a 3' blocking group was hybridized to the poly d(T) tail of the purified cDNA. Double-stranded T7-promoter was synthesized at room temperature for 30 minutes using Klenow fragment of DNA Polymerase I (Invitrogen). The 3' blocker was used to prevent the synthesis of complete second strand cDNA during the T7 promoter "fill-in" reaction. Excess T7 promoter template was removed using the MinElute PCR Purification Kit. The purified cDNA, which contained a double-stranded T7 promoter linked to a single stranded cDNA template, was used for *in vitro *transcription using the MegaScript kit (Ambion). Amplified sense RNA was purified using the RNeasy Kit (Qiagen) and the manufacturer's recommendation for RNA clean up.

### cDNA synthesis

Five μg of each amplified RNA sample and unamplified control (with the exception of the NuGen Ovation amplification which yielded cDNA directly) was reverse transcribed into cDNA. RNA was added to 1 μl of 50 ng/μl random hexamer primer, 1 μl 10 mM dNTP mix (Sigma, St. Louis MO), and RNase-free water to make 12 μl. The mixture was denatured at 65°C for 5 min and immediately chilled. Reaction buffer and SuperScript II (Invitrogen) was then added and the volume was adjusted to 20 μl. The mixture was then incubated at 25°C for 10 minutes, 42°C for 50 minutes, and finally 70°C for 15 minutes to stop the reaction.

### Quantitative real-time PCR (qPCR)

Each treatment assay was conducted across a total of four 384-well plates per assay. Each plate targeted 48 different genes for PCR amplification, each with 3 brain and 3 liver samples as well as 6 GAPDH wells for normalization across plates. A calibrator plate was used for each treatment to determine the concentration of cDNA required from each amplification technique to produce a GAPDH C_t _comparable to that derived from the 1:10 dilution in the unamplified control. cDNAs for the NuGen, 2 rounds of SenseAmp version 1–2, and SenseAmp version 1–2 on degraded RNA were all diluted 100X. cDNAs from SenseAmp version 1 were diluted 120X. The remaining version 2, version 1–2, and messageAmp cDNA samples were diluted 200X. 2 μl of diluted cDNA was added to the primer pair mix and SYBR Green Master Mix (Applied Biosystems) in each well. qPCR was conducted on the Prism 7900HT Sequence Detection System (Applied Biosystems). Plates were run for 40 cycles and fluorescence intensity measured after every cycle. For each target sequence the average cycle number at which fluorescence was detected above background in the exponential phase of amplification was obtained for the brain and liver samples. This value, C_t_, or cycle number at threshold, was used for calculations of relative abundance of mRNA molecules in the liver samples compared to the brain samples for each of the amplification methods.

### PCR primer efficiency

The efficiency of each of the 192 target gene PCR primer pairs was calculated using the LinRegPCR software [22]. Normalized fluorescence values for each well were recorded for each cycle of RT-PCR. LinRegPCR used the log of these data to calculate the linear regression of a "window of linearity" in the exponential phase of amplification. The efficiency of the primer pairs corresponds to 10^slope ^of the linear regression of the normalized log fluorescence values within the "window of linearity" for each well. As per the recommendations for this calculation, PCR primer pairs with strongly deviating PCR efficiencies or correlation coefficients below 0.999 were discarded [22] Average efficiency values for each primer pair were determined and used in equation (1) to reveal the relative abundance of mRNA in each sample.

### Data analysis

The following algorithm was applied to the results from each amplification method as well as the unamplified control. As per the ΔΔC_t _qPCR analysis method, an average cycle number was determined at which fluorescence crossed a threshold above background. The resulting C_t _value was recorded for each tissue type and target gene. This C_t _value was normalized across plates by subtraction of the C_t _value from the housekeeping gene GAPDH. This value represents the ΔC_t_. The ΔC_t _from the reference brain samples was subtracted from the ΔC_t _obtained from the liver samples. This gave the change in ΔC_t _between the two tissues or ΔΔC_t_. With the addition of calculated primer pair efficiencies, the ratio of gene expression for each target mRNA sequence between the two tissues was determined using equation (1). Ratios of expression values for liver tissues relative to brain (RQ) for each amplification technique were plotted in Excel (Microsoft, Redmond WA) and Pearson correlations to the unamplified control were determined using Analyze-It (Analyse-It Software, ), an Excel Statistics Add-on.

## Authors' contributions

LAG performed all qPCR assays, selected and designed primer pairs, developed databases holding all data, and prepared the first draft of the manuscript. JB, JS, KH and RCG participated in the design of the study, developed the SenseAmp reactions, and modified the reactions in response to qPCR validation results. RCG supervised SenseAmp development. RPH conceived the study, supervised the qPCR assays, and coordinated work. All authors read and approved the final manuscript.
